# The essential role of bursicon during *Drosophila *development

**DOI:** 10.1186/1471-213X-10-92

**Published:** 2010-08-31

**Authors:** Brandon J Loveall, David L Deitcher

**Affiliations:** 1 Department of Neurobiology and Behavior, Cornell University, Ithaca, NY 14853, USA

## Abstract

**Background:**

The protective external cuticle of insects does not accommodate growth during development. To compensate for this, the insect life cycle is punctuated by a series of molts. During the molt, a new and larger cuticle is produced underneath the old cuticle. Replacement of the smaller, old cuticle culminates with ecdysis, a stereotyped sequence of shedding behaviors. Following each ecdysis, the new cuticle must expand and harden. Studies from a variety of insect species indicate that this cuticle hardening is regulated by the neuropeptide bursicon. However, genetic evidence from *Drosophila melanogaster *only supports such a role for bursicon after the final ecdysis, when the adult fly emerges. The research presented here investigates the role that bursicon has at stages of *Drosophila *development which precede adult ecdysis.

**Results:**

We addressed the mechanism and timing of hormonal release from bursicon-positive motor neurons at the larval neuromuscular junction. Our findings indicate that vesicle membrane proteins which are required for classical neurotransmitter release are also expressed at these peptidergic motor neuron terminals; and that these terminals secrete hormones including bursicon at the neuromuscular junction, coinciding with larval ecdysis. This release surprisingly occurs in two waves, indicating bursicon secretion preceding and following the ecdysis sequence. Next, we addressed the functional significance of bursicon signaling during development, by disrupting the expression of its receptor, rickets, in different target tissues. We determined that rickets is developmentally required in the epidermis and imaginal discs for proper formation of the prepupa. It is also required to harden the pharate adult cuticle before eclosion. Significantly, we have also found that the available rickets mutants are not genetic nulls as previously believed, which necessitated the use of targeted RNA interference to disrupt rickets expression.

**Conclusions:**

Our results are consistent with the view that bursicon is the insect tanning hormone. However, this is the first study to rigorously demonstrate both its release and function during development. Importantly, we provide new evidence that bursicon release can precede the initiation of larval ecdysis, and that bursicon tans the puparium. Our results firmly establish bursicon signaling as essential to insect growth and development.

## Background

For all their remarkable diversity, all insects are faced with a recurring problem during their development: the replacement of a constricting exoskeleton after periods of intermolt growth. The successful solution to this problem is to form a new, larger cuticle beneath the hardened exoskeleton. Shedding of the older exoskeleton can only occur once the new cuticle is complete. The cuticle-shedding behaviors collectively known as ecdysis are orchestrated by a suite of interacting peptide hormones including ecdysis-triggering hormone (ETH), eclosion hormone (EH) and CCAP (reviewed extensively in [1-3]). The canonical model proposes that ETH and EH coupled release initiates the preparatory behaviors of ecdysis; CCAP terminates these early behaviors and also serves to trigger the final bouts of shedding the exuvia [4].

Upon completion of their final ecdysis (eclosion), adult *Drosophila *emerge with a soft cuticle and unexpanded wings. While these features are advantageous for confinement within an otherwise too small puparium, they are not adaptive to life outside of the puparium. Instead, shortly following eclosion the wings expand to their full size and the new cuticle undergoes sclerotization and melanization (tans) (see [1] for a recent review).

Several key observations provide evidence that these post-eclosion events are regulated by the heterodimeric neuropeptide bursicon through its receptor, rickets. First, the *rickets *mutants *rk^1 ^*and *rk^4 ^*are incapable of wing expansion behaviors or tanning their cuticle, even when challenged with hemolymph extracts containing bursicon from newly eclosed wild-type flies [5]. Similarly, *bursicon *mutants are unable to successfully execute this phase of post-eclosion development [6]. Bursicon hormone has been shown to activate the rickets receptor, by increasing downstream cAMP activity [7,8], completing the link between hormone signaling and post-eclosion development.

Establishing a role for the bursicon signaling pathway preceding eclosion has been more difficult to demonstrate, primarily because *rk^1 ^*and *rk^4 ^*exhibit few if any developmental defects prior to eclosion (cf. [5]). However, we wondered if the bursicon signaling pathway performs similar cuticle tanning roles following larval and pupal ecdyses. In support of this possibility, transcripts of *rk *and the bursicon subunits *burs *and *pburs *are found at all stages of post-embryonic development [7,8] (summarized in [9]). Furthermore, BURS subunit immunoreactivity is present in several larval tissues, including CNS [7,10] and peripheral motor neuron terminals [10]. Recent evidence has also shown that a subset of CCAP neurons which co-express bursicon undergo Ca^2+ ^changes in their cell bodies during pupal ecdysis (in response to exogenous ETH), suggesting that these neuropeptides are secreted at this point in ecdysis [11]. Notably, these same neurons have axonal projections with 'type III' boutons at the neuromuscular junction (NMJ) [12]. Thus, it is likely that bursicon is released contemporaneously with ecdyses other than eclosion, although no study has quantified the secretion of any neuropeptide from type III boutons in larval or pupal stages. The expression of bursicon and its likely release during development are strong indicators of additional functions apart from the post-eclosion events which it is known to regulate. However, these may not be easily deducible with the available *burs *and *rk *mutants, none of which result in a complete absence of gene product (cf. [5,6]).

On the other hand, several transgenic studies hint at the possibility that disrupting bursicon signaling during development can have lethal consequences. Ectopic expression of the cell death gene *reaper *in CCAP neurons [13] (some of which express bursicon) results in two classes of progeny: those flies which eclose exhibit cuticular deformities and unexpanded wings remarkably similar to *burs *and *rk *mutants. The other class of progeny are incapable of performing head eversion, a crucial event at pupal ecdysis, and subsequently die as pupae. The defects at head eversion are even more frequent when CCAP neuron membrane excitability is suppressed by transgenic expression of a human K^+ ^channel inward rectifier (UAS-Kir2.1) [14]. Nevertheless, this pupal lethal phenotype should be interpreted with the caveat that CCAP neurons express multiple neuropeptide identities, including CCAP, bursicon and myoinhibitory peptide [11], which may obscure the cause of the head eversion defect. Thus, neither available genetic mutants nor cell ablation studies are a reliable means to elucidate the function of bursicon signaling during development.

Alternatively, the function of bursicon/rickets in developing life stages could be examined with RNA interference (RNAi). This method takes advantage of endogenous cellular mechanisms to recognize double-stranded RNA (dsRNA) and abolish any identical native transcripts: by introducing transcript-specific dsRNA, the expression of a gene of interest can be silenced [15]. Surprisingly, this technique has not yet been used to experimentally manipulate *burs *or *rk *expression in *Drosophila*.

The hormone bursicon (acting through its receptor, rickets) is a key factor in tanning the new cuticle. However, direct and incontrovertible genetic evidence for the functional role of this signaling pathway has only been demonstrated in post-eclosion *Drosophila*. The lack of any pre-eclosion defects in *burs *and *rk *mutants may be because these mutations are hypomorphs, or alternatively because the bursicon signaling pathway is not functionally relevant prior to eclosion. To address these distinct possibilities, we examined bursicon's peripheral release from type III boutons during the 2^nd ^larval and pupal ecdyses. The developmental role of bursicon was also examined by manipulating expression of both *bursicon *and *rickets*, via targeted RNAi. Our results show that bursicon is released during the 2^nd ^larval ecdysis, and strongly suggest that it is released during pupal ecdysis as well. We also show that *bursicon *and *rickets *expression are essential during pupal development for viability.

## Results

### Bursicon expression at the larval NMJ is limited to type III boutons

In *Drosophila*, bursicon is produced in a subset of CCAP neurons in the CNS [6,9,14]. Among this subset, the neuromeres in T3 (which express BURS but not PBURS [7]) and A1-A4 send motor neuron axons to the type III boutons of NMJ 12 and 13 [12,16]. Since CCAP and bursicon share overlapping expression patterns in the ventral ganglion, it is likely that bursicon is also co-expressed in type III boutons.

**Figure 1:**
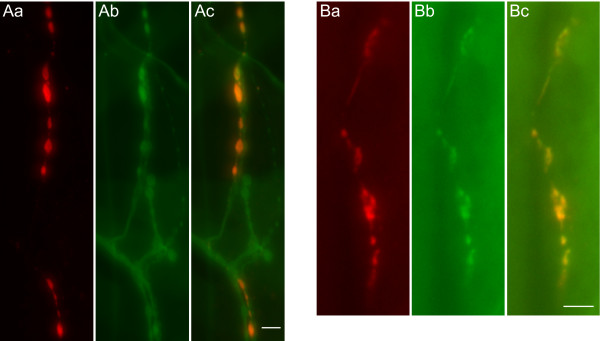
**Bursicon is expressed in type III boutons at the larval NMJ**. Bursicon immunoreactivity (BURS-IR) has previously been shown in the periphery [10], without confirming its distribution pattern. (Aa) BURS-IR at a representative muscle 12 at the larval NMJ. (Ab) HRP immunoreactivity labels all bouton types at NMJ 12. (Ac) Bursicon distribution is found in some, but not all boutons at NMJ 12. (B) To verify if bursicon is expressed in type III boutons, we used transgenic larvae expressing the fluorescent neuropeptide marker ANF-EMD with a CCAP-GAL4 promoter. (Ba) BURS-IR at a representative muscle 12. (Bb) Vesicles with ANF-EMD are distributed in type III boutons. (Bc) BURS co-localizes with the ectopic ANF-EMD marker. Scale bars = 10 μm. An antibody which recognizes the bursicon-α subunit was consistently used for BURS-IR.

Immunoreactivity to the bursicon subunit BURS (BURS-IR) at a representative larval muscle 12 stained with HRP illustrates that bursicon is present in some but not all boutons at NMJ 12 (Figure 1A), which underscores the need to determine which boutons express bursicon. Whereas peripheral bursicon immunoreactivity (BURS-IR) has previously been demonstrated in both larval and adult preparations [10], its presence in a specific bouton type has not been independently established. To examine whether bursicon is expressed in type III boutons, we looked for its co-localization with an exogenous GFP marker driven by CCAP-GAL4. In this case, an emerald-GFP tagged atrial natriuretic factor (or UAS-ANF-EMD) construct [17] was used as the marker for type III boutons. Our results indicate that BURS-IR co-localized exclusively with ANF-EMD in type III boutons (Figure 1B), where the CCAP-GAL4 pattern has been previously shown [12].

### Expression of vesicle membrane proteins in type III boutons

**Figure 2:**
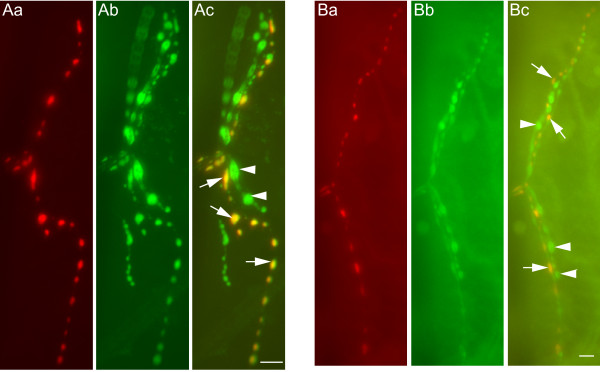
**Bursicon co-localizes with the pre-synaptic markers N-SYB and CSP at the NMJ**. (A) Neuronal synaptobrevin (N-SYB) expression at NMJ 12 includes type III boutons. (Aa) BURS-IR in type III boutons. (Ab) Distribution of N-SYB-IR at NMJ 12. (Ac) N-SYB is expressed in multiple bouton types, including type III. (B) Cysteine string protein expression pattern at NMJ 12 includes type III boutons. (Ba) BURS-IR as a marker for type III boutons. (Bb) CSP-IR labels multiple boutons at NMJ 12. (C) BURS and CSP patterns co-localize in type III boutons. The expression of CSP immunoreactivity in type III boutons appears to be weaker than in other boutons at NMJ 12. In (A) and (B), arrows indicate representative boutons that co-express BURS and the relevant pre-synaptic marker, whereas arrowheads indicate boutons that do not express bursicon. Scale bars = 10 μm.

We were curious whether the bursicon expressed in type III boutons represents a releasable pool of vesicles. To answer this question, we examined the expression of two pre-synaptic vesicle protein markers in type III boutons, neuronal Synaptobrevin (N-SYB) and cysteine string protein (CSP). Double-labeling of NMJ preparations with bursicon (Figure 2Aa) and N-SYB antibodies (Figure 2Ab) demonstrates that N-SYB is expressed in type III boutons (Figure 2Ac; arrows), as well as boutons which are devoid of BURS-IR (Figure 2Ac; arrowheads). Similarly, BURS-IR (Figure 2Ba) and CSP-IR (Figure 2Bb) co-localize in type III boutons (Figure 2Bc; arrows), with CSP-IR also occurring outside of the BURS-IR pattern (Figure 2Bc; arrowheads). The presence of N-SYB and CSP in type III boutons is consistent with these vesicles being a releasable pool of secretory granules.

### Secretion of vesicles from type III boutons before and after larval ecydsis

The observation that the vesicle membrane proteins N-SYB and CSP are expressed in type III boutons prompted us to examine if vesicle release occurs during the larval stage. Aside from bursicon, hormones such as CCAP and MIP are expressed in these peptidergic terminals, and have roles in ecdysis and post-ecdysis events. Thus, hormone release from the type III boutons may be contemporaneous with ecdysis. To examine vesicle release in a time window corresponding to ecdysis, we decided to image live transgenic larvae that expressed UAS-ANF-EMD in the CCAP-GAL4 pattern. The ANF-EMD construct has been used successfully in several studies to monitor vesicle release from secretory cells during endogenous behaviors [18-21].

**Figure 3:**
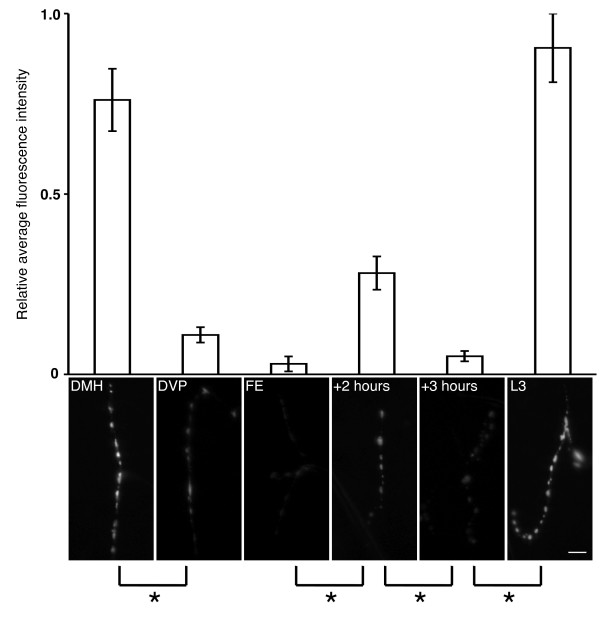
**Two phases of vesicle release from type III boutons overlap with larval ecdysis**. We observed fluorescence changes in the type III boutons of CCAP>ANF-EMD larvae. Six stages were chosen that broadly extend over the duration of the 2^nd ^larval ecdysis: 'DMH', which precedes the onset of ecdysis; 'DVP', which coincides with the initiation of ETH release; 'FE', which signifies the completion of ecdysis; '+2 hours' after FE; '+3 hours' after FE; and 'L3', wandering 3^rd ^instar larvae. For DVP, we selected animals at the earliest point when the new vertical plates were recognizable. At each stage, the fluorescence intensity of all visible type III boutons from 4 animals was measured and converted to fluorescence intensity averages (see Methods), in arbitary units (top panel). Representative type III boutons from each stage are shown below the corresponding fluorescence intensity measurements with accompanying asterisks representing single-factor ANOVA results for consecutive stages (bottom panels). The results significantly show two waves of ANF-EMD release, before DVP and after FE, as shown by an asterisk. For *, p < 0.005. Error bars indicate +/- SEM. Scale bar = 10 μm.

While not directly addressing the identity of endogenous hormone release, this technique allowed us to accurately quantify when the type III boutons release ANF-EMD, by directly measuring fluorescence intensity fluctuations (Figure 3). We initially examined NMJs T3 and A1-A4 of CCAP>ANF-EMD animals approaching the second larval ecdysis, and those which had begun secreting ETH, regarded as the initial peptide hormone trigger of ecdysis behaviors [22]. These two stages can be distinguished on the basis of mouthpart morphology known as 'double mouth hooks' (DMH) and 'double vertical plates' (DVP), respectively [22]. Imaging of the DMH stage indicated high levels of ANF-EMD in type III boutons. Less than two hours later, at the earliest recognizable DVP time, there is an 85% decrease in fluorescence intensity, indicating large-scale secretion. This is a startling result, since no ecdysis-related peptide hormone is currently known to precede the release of ETH at the DVP stage. This wave of ANF-EMD release continues through the end of ecdysis behaviors, until when the 3^rd ^instar larva finally breaks through the old cuticle with a thrusting forward escape (FE) motion [22]. The level of ANF-EMD release at FE represents its lowest expression level, a 96% drop in fluorescence intensity from the initial DMH levels. Following this stage, type III boutons appear to refill with ANF-EMD. Measurements taken two hours after FE have rebounded to a level representing 37% of initial DMH levels, suggesting that refilling has begun (see +2 hours, Figure 3). However, within one hour we detected a second decline in fluorescence intensity in type III boutons: at three hours post-FE (see +3 hours, Figure 3) approximately 82% vesicle release has occurred, relative to the preceding stage. Thus there appears to be two distinct waves of ANF-EMD release from type III boutons, the first preceding ETH release at DVP and the second following the completion of ecdysis behaviors at FE. By the wandering 3^rd ^instar stage, the fluorescence intensity has returned to levels seen before ecdysis (see L3, Figure 3). Single-factor ANOVA tests indicate significant differences between the following stages of larval ecdysis: DMH to DVP (p = 0.004); FE to +2 hours (p = 0.0008); +2 hours to +3 hours (p = 0.001); and +3 hours to L3 (p = 3.4 × 10^-5^). The one stage comparison without a significant difference is DVP to FE (p = 0.08). As a control we also examined larvae expressing ANF-EMD with a pan-neural driver, elav-GAL4. Examination of normally non-peptidergic type I boutons on NMJ 6/7 between DMH and DVP revealed no detectable change in fluorescence intensity in elav>ANF-EMD animals (data not shown). This indicates that the changes in the type III boutons are not the result of large-scale synaptic activity at the NMJ.

To confirm whether bursicon is released at a stage which precedes ETH release, we dissected wild-type larvae into fillets and tracheae. The larval fillets were stained with anti-BURS, whereas the tracheae (which contain the ETH-secreting Inka cells) were stained with anti-ETH. Between DMH and DVP stages, we detected that BURS-IR significantly decreased by 57% in type III boutons (see Additional file 1). This period corresponds with the first wave of release detected in CCAP>ANF-EMD live preparations (see Figure 3). In the corresponding tracheae from these animals we were unable to observe a change in ETH-IR between DMH and DVP (see Additional file 1), confirming that ETH (the trigger for ecdysis behaviors) has not yet been released. Together, these data support our claim that a wave of bursicon release precedes ETH release, at the 2^nd ^larval ecdysis.

### Secretion of vesicles from type III boutons during pupal ecydsis

**Figure 4:**
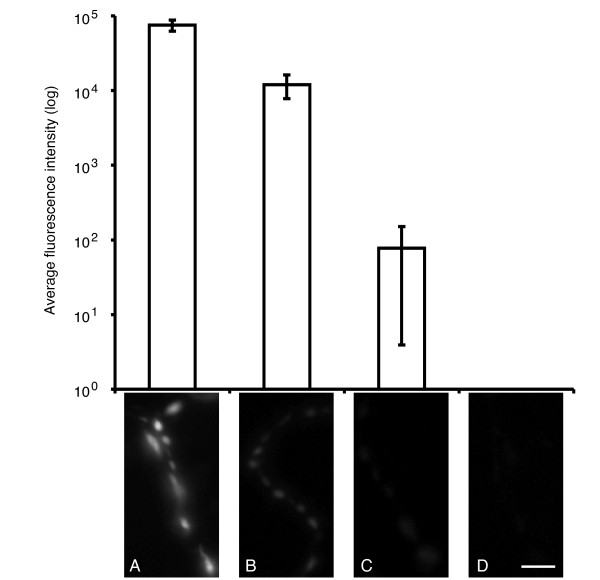
**Hormone release from type III boutons at the pupal NMJ coincides with pupal ecdysis**. Changes in fluorescence intensity are also observed in type III boutons of CCAP>ANF-EMD animals at pupal ecdysis. (A) P3 stage pupa, preceding pupal ecdysis by several hours. (B) Pupa at end of P4(i) stage, shortly after initiation of the ecdysis sequence. (C) P4(ii) stage pupa immediately following head eversion (occurring during pupal ecdysis). (D) P5(i) pupa following elongation of legs, approximately 1/2 hour after head eversion. Average fluorescence intensity is reported on a log scale. For (D), no detectable fluorescence intensity is reported. Identification of pupal stages is described in the Methods. Error bars indicate +/- SEM. Scale bar = 10 μm.

We wondered whether at other ecdyses if bursicon release can precede the initiation of ecdysis behaviors. A previous study showed that Ca^2+ ^levels rose in CCAP neurons at pupal ecdysis, in response to ETH [11]. Using morphologically staged pupae (from P3 through P5(i)), we examined this period for ANF-EMD release from type III boutons (Figure 4). The P3 pupal stage can precede pupal ecdysis by several hours, and here we observed the highest levels of ANF-EMD fluorescence intensity (A in Figure 4). Within minutes of ETH release, the ecdysis sequence begins with pre-ecdysis behaviors, recognized as the eviction of an air bubble in the posterior pupal space [11]. This time-point corresponds to our staged P4(i) pupae, in which we observed ANF-EMD fluorescence levels at the NMJ begin to decline, indicating hormone release from type III boutons (B in Figure 4). ANF-EMD fluorescence continues to decline through the completion of head eversion, which shortly follows initiation of ecdysis behaviors (cf. [11]); this time-point corresponds to our staged P4(ii) pupae (C in Figure 4). After the completion of ecdysis behaviors and leg extension, ANF-EMD fluorescence is almost absent from type III boutons in P5(i) pupae (D in Figure 4). Our results in Figure 4 are consistent with large Ca^2+ ^increases observed in CCAP neurons during the pupal ecdysis sequence (see [11]). However, since we used the appearance of the posterior air bubble to define P4(i) pupae, we are unable to make any claims about the exact timing of ANF-EMD release as it relates to ETH secretion, as by this time ETH secretion has already begun [11].

### Ubiquitous expression of UAS-burs RNAi obstructs advancement beyond the pharate adult stage

**Figure 5:**
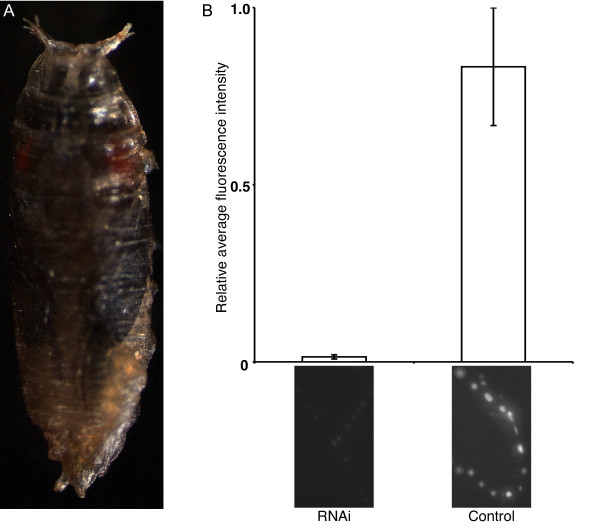
**Knock-down of burs transcripts by RNAi impedes progression to the pharate adult stage**. (A) Development of Act5C>burs RNAi animals proceeds until the pharate adult stage, when all progeny die trapped within the puparium. (B) BURS-IR comparisons of Act5C>burs RNAi larvae and driver-less UAS-burs RNAi control larvae confirms that ubiquitous expression of burs RNAi severely limits the expression of bursicon at the NMJ, as quantified by fluorescence intensity. For each data point, larval NMJs (n = 16) from two animals were observed. Representative 'RNAi' and 'Control' NMJs accompany the data points (in lower panels). Error bars indicate +/- SEM.

In the previous sections, we found evidence for release of bursicon from type III boutons during development. We next wished to perturb its expression in order to probe its developmental role. Since *burs *mutants are viable but do not show an observable mutant phenotype until post-eclosion (cf. [6]), we chose to affect *burs *expression by RNA interference (RNAi). Larval development apparently proceeds unimpeded when UAS-burs RNAi is ubiquitously expressed (Act5C>burs RNAi). However, pharate adults of this genotype were unable to escape from their puparia (Figure 5A). Although opened opercula were occasionally observed, eclosion was still not possible from the puparium. To confirm a knock-down effect by UAS-burs RNAi, we labeled Act5C>burs RNAi larval NMJs with anti-BURS antiserum and saw a dramatic decrease in fluorescence intensity, relative to a UAS-burs RNAi control (with no Act5C-GAL4) (Figure 5B). As an additional control, we saw no change in CCAP-IR in similar Act5C>burs RNAi tissue preparations (data not shown).

### Ubiquitous expression of UAS-rk RNAi arrests development

**Figure 6:**
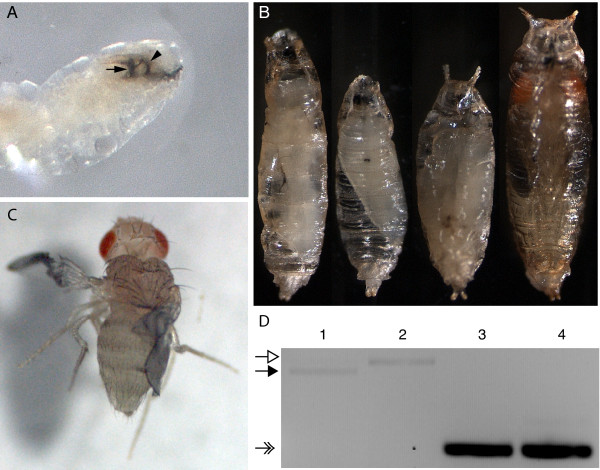
**Lethality results from ubiquitous expression of UAS-rk RNAi**. (A) Within standard food vials, Act5C>rk RNAi progeny all die before the end of the 3^rd ^larval stage. These larvae often exhibit the double vertical plates phenotype, indicative of failed larval ecdysis. New 3^rd ^instar mouthparts are labeled with the arrow; the 2^nd ^instar mouthparts which failed to shed are labeled with the arrowhead. (B) If Act5C>rk RNAi larvae are rescued from food vials and allowed to develop on grape juice agar plates, they progress to the pupal stage. Lethality is 100% during this stage, but the timing and phenotype are highly variable. Note the pale color and flattened shape of the puparia. All pupae are shown at the same scale, from the ventral side. (C) A second Act5C-GAL4 stock was also used, referred to here as Act5C(II). Although most Act5C(II)>rk RNAi progeny die as pupae (data not shown), adults occasionally eclose. Their wings never expand and their cuticle never tans. Panels A, B, and C are not shown at the same scale. (D) RT-PCR with pupal cDNA templates was performed to confirm that ubiquitous expression of UAS-rk RNAi knocks down *rk *transcript levels. Lanes 1 and 3: UAS-rk RNAi (control with no driver). Lanes 2 and 4: Act5C>rk RNAi. Open arrow: expected genomic band size for *rk*. Closed arrow: expected cDNA band size for *rk*. Note the absence of an appropriate sized *rk *cDNA band for Act5C>rk RNAi in lane 2. Feathered arrow: expected cDNA size for the positive control, *RpS26*.

To further explore the functional significance of the bursicon pathway during development, we next examined the role of its receptor, rickets. We chose to address its role by expressing rk RNAi widely and screening for resultant phenotypes. In standard growing conditions within food vials, ubiquitous expression of UAS-rk RNAi (Act5C>rk RNAi) results in progeny that are unable to develop beyond the larval stage, and the few 3^rd ^instar larvae which we recovered often died with a 'double vertical plates' phenotype (Figure 6A), indicative of failed larval ecdysis [22]. However, if larvae of this genotype are rescued from the food and allowed to mature on grape juice agar plates, progeny can develop until the pupal stage, at which point a range of lethal phenotypes is observed (Figure 6B), including pupae that resemble larvae in shape, and pupae that arrest before or shortly after pupal ecdysis. These pupae appear pale in color and are extremely flimsy, suggesting that the puparia have not "tanned". Pharate adults are also observed (see Figure 6B), but they always fail to eclose.

We also examined the effect of the same UAS-rk RNAi construct when driven by a different Act5C-GAL4 driver (referred to here as Act5C(II)-GAL4). These progeny primarily die at the pupal stage (data not shown). The few progeny which eclose resemble *rk *mutants in that they are unable to tan their cuticle or expand their wings (Figure 6C). Contrary to classic *rk *phenotypes, these flies have great difficulty standing. This is likely a consequence of extreme cuticular defects, and all adults die within 24 hours of eclosion. The lethal phenotypes which result when UAS-rk RNAi is driven with either Act5C-GAL4 are consistent with a previous report that the same UAS-rk RNAi construct is 100% lethal when ubiquitously expressed with Act5C-GAL4 [15]. However, these RNAi phenotypes were not described, nor was the underlying basis explored. Thus, expression of UAS-rk RNAi with either Act5C driver results in pupal lethality.

To confirm that the UAS-rk RNAi construct does indeed knock down *rickets *transcript levels, we performed RT-PCR on cDNA from pupae of both Act5C>rk RNAi and UAS-rk RNAi (Figure 6D). The results show that for the expected size of amplified *rk *cDNA, *rk *transcript levels are undetectable in Act5C>rk RNAi as compared to the UAS-rk RNAi control.

### The *rickets *mutations *rk^1 ^*and *rk^4 ^*are more severe as hemizygotes

The homozygous *rickets *mutants *rk^1 ^*and *rk^4 ^*have previously been described as null mutants [5]. Consequently, gene silencing by ubiquitous RNAi expression should not result in a mutant phenotype that is more severe than expected from a genetic 'loss of function' mutation. Surprisingly, the phenotypes documented in Figure 6 contradict this basic principle in two ways. First, the classic mutant alleles are perfectly viable as homozygous adults, as compared to Act5C>rk RNAi (refer to Figures 6A and 6B). Second, although cuticle deformations are a hallmark of *rickets *mutations, neither mutant allele is crippled to the extent of Act5C(II)>rk RNAi adults (refer to Figure 6C). This unanticipated disparity in phenotypes suggests that either ubiquitous expression of UAS-rk RNAi has unintended consequences in addition to silencing of the *rickets *gene, or that *rk^1 ^*and *rk^4 ^*alleles do not truly represent genetic null mutants.

**Figure 7:**
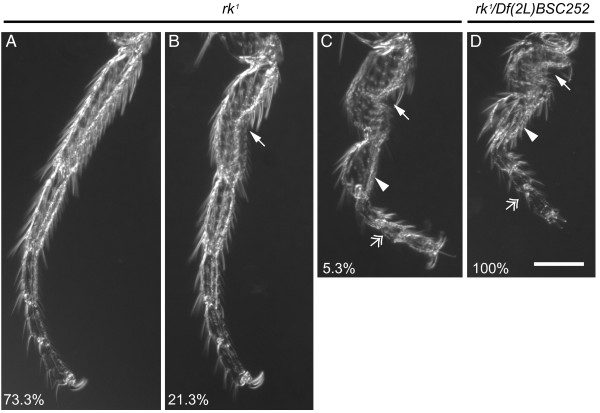
**Analysis of leg phenotypes reveals that *rk^1 ^*is not a null mutant**. The homozygous *rk^1 ^*stock displays incomplete penetrance of leg deformities. Leg deformities in *rk^1 ^*homozygotes are classified as resembling wild-type legs (A), moderately kinked (B), or severely kinked (C). A moderate leg deformity is defined by the presence of a kink in the first tarsal segment (arrow). Severe leg deformities additionally exhibit a bulbous tarsal segment (arrowhead) and a rotated tarsal segment (feathered arrowhead). The corresponding percentage of leg deformities is given (A-C) for a random sample of adult metathoracic legs from the *rk^1 ^*stock (n = 150). (D) When hemizygous, 100% of *rk^1^/Df(2L)BSC252 *animals exhibit the most severe leg deformities (n = 64). This enhanced penetrance indicates that the *rk^1 ^*allele is not a null. Scale bar = 0.1 mm.

The *rk^1 ^*and *rk^4 ^*mutants were originally defined as nulls on the basis that the heteroallelic combination of two distinct cytologically defined deletions which incompletely overlap with *rickets *is indistinguishable from homozygous *rk^1 ^*and *rk^4 ^*adults [5]. Since the deficiencies used are no longer available, we were unable to assay these alleles in this fashion. To test if the *rk *alleles are homozygous nulls, we created hemizygotes by crossing either *rk^1 ^*or *rk^4 ^*to *Df(2L)BSC252/CyO*. Importantly, the *Df(2L)BSC252 *deficiency contains molecularly defined breakpoints which delete *rk *in its entirety [23] (unlike the deficiencies used in earlier studies). In this scenario, hemizygous mutants, whether *rk^1^/Df *or *rk^4^/Df*, can only display a more severe mutant phenotype than homozygous *rk^1 ^*and *rk^4 ^*flies if these are not loss of function alleles. To test this hypothesis we had to select a suitable trait that might be enhanced when *rk *is hemizygous with *Df(2L)BSC252*. For example, the complete penetrance of unextended wings in *rk^1 ^*and *rk^4 ^*makes this trait uninformative to score in a loss of function test. Indeed, the wings of both *rk^1^/Df *and *rk^4^/Df *flies are indistinguishable from the respective homozygous mutant alleles (data not shown). Another aspect of the *rickets *mutant phenotype occurs with reduced penetrance: both *rk^1 ^*and *rk^4 ^*flies can exhibit a range of leg deformities including bowed femora and flattened tarsal segments [5]. We hypothesized that the reduced penetrance of leg deformities in homozygous mutants could be enhanced in hemizygous mutants. To determine the severity of *rickets *disruption, we scored deformities in metathoracic legs of both homozygous and hemizygous *rk^1 ^*and *rk^4 ^*flies. In a random sampling of homozygous *rk^1 ^*legs (n = 150), 73.3% are normal, 21.3% exhibit moderately kinked tarsi, and only 5.3% have severely kinked legs (Figure 7). Remarkably, 100% of *rk^1^/Df *flies have severe tarsal deformities, representing a greater-than 19-fold increase. A less drastic transformation was seen in our parallel *rk^4 ^*study: whereas 100% of homozygous *rk^4 ^*legs have tarsi with no defects (n = 106), 2/3 of legs from *rk^4^/Df *flies (n = 42) were scored with tarsal deformities (data not shown).

A number of uneclosed *rk^1^/Df *progeny were also observed. Could developmental lethality be affected in *rk^1 ^*hemizygotes? We addressed this question by crossing *rk^1 ^*to *Df(2L)BSC252/CyO, ActGFP*, to distinguish between heterozygous and hemizygous *rk^1 ^*pupae. As expected, roughly equal numbers of *rk^1^/Df *(n = 53) and *rk^1^/CyO, ActGFP *pupae (n = 47) were obtained. However, only 60.4% (n = 32) of *rk^1^/Df *flies eclosed, as compared to 95.7% (n = 45) of *rk^1^/CyO, ActGFP*. In a parallel study, we compared developmental lethality in homozygous *rk^1 ^*and heterozygous *rk^1^*/+ flies. In this case, crossing the homozygous *rk^1 ^*stock to *rk^1^*/+ results in similar numbers of viable *rk^1 ^*(n = 38) and *rk^1^*/+ (n = 37) adult flies, and no uneclosed pupae. These two experiments bolster our claim that *rk^1^/Df *is indeed a stronger disruption of rickets function than homozygous *rk^1 ^*by itself. The decreased eclosion rate in *rk^1^/Df *appears to be due to an inability in otherwise healthy looking pharate adults to escape from the puparium.

We conclude that *rk^1 ^*does not represent a null allele when leg morphology and developmental lethality are taken into account. Therefore it is not unreasonable for Act5C>rk RNAi to display more severe phenotypes than the available homozygous *rk *mutant alleles.

### *rickets *expression is required in developing epidermal tissue and imaginal discs

As a prelude to manipulating *rickets *expression by RNAi with more selective GAL4 drivers, we asked where rickets might normally be expressed during development. A recent large-scale gene expression study has identified *rickets *transcripts in several tissues during development by microarray [24]. In their study, two larval tissues had higher levels of *rk *transcripts: the CNS and the fat body. In addition to these tissues, we reasoned that since bursicon acts on the epidermis to tan the cuticle (see [9] for review), *rk *may be expressed developmentally in epidermal tissue, or in tissue that eventually forms adult structures (i.e., the imaginal discs). These data suggest that targeted UAS-rk RNAi expression in separate tissues could reveal developmental requirements for *rickets *expression, as assayed by their mutant phenotypes. To test this hypothesis, we crossed UAS-rk RNAi to neural (elav, n-syb), peptidergic (CCAP, c929, 386, 36 y) and fat body (r4, FB) GAL4 lines. With the above GAL4 drivers, expression of rk RNAi generated flies that appeared healthy and indistinguishable from wild-type flies (data not shown). We conclude that rk RNAi in CNS, peptidergic neurons, or fat body alone is insufficient to generate *rickets*-like phenotypes.

**Figure 8:**
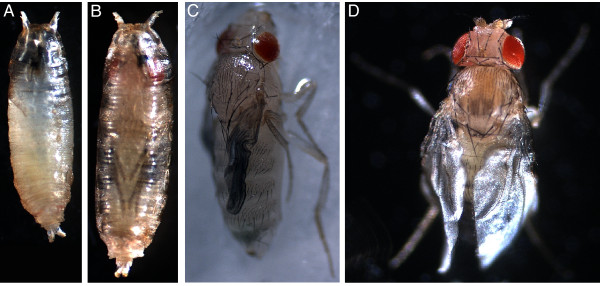
**Phenotypes resulting from different GAL4 drivers of UAS-rk RNAi**. (A) All T76>rk RNAi progeny die with small, soft, untanned puparia before pupal ecdysis. The puparia often have a slight crescent bend to them, rather than a straight orientation along the anterior-posterior axis. (B) In contrast, T80>rk RNAi develop into pharate adults, but all are then unable to eclose from their puparia and die. (C) A limited number of 69B>rk RNAi flies are able to eclose, but their legs are flaccid and collapse under the weight of the body. All flies die within 24 hours, before ever expanding their wings or tanning their cuticle. (D) C855a>rk RNAi flies successfully eclose without any obvious problems. However, all adults have drooping, partially extended wings. This appears to be a result of the successful deployment of wing expansion behaviors in the absence of cuticular tanning. Only A and B are shown at the same scale, to emphasize the smaller size of T76>rk RNAi pupae.

In contrast, expression of UAS-rk RNAi by GAL4 lines with reported epidermal and/or imaginal disc expression patterns (see [25,26]) produced several different mutant phenotypes. The observed rk RNAi phenotypes were dependent on the specific GAL4 driver. The resulting animals ranged from those unable to complete the prepupal stage, to healthy adult flies. For example, UAS-rk RNAi expression with T76-GAL4 results in 100% lethality prior to pupal ecdysis. From this genotype we only observed animals in small, misshapen and untanned puparia (Figure 8A). Two separate drivers, T80-GAL4 and 69B-GAL4, give nearly identical results with UAS-rk RNAi, though less severe than with T76-GAL4. All T80>rk RNAi and most 69B>rk RNAi progeny die as pharate adults (Figure 8B). Apart from their failure to eclose, these progeny appear otherwise normal. Occasionally, adult 69B>rk RNAi "escapers" are observed (Figure 8C). Their untanned cuticle and unexpanded wings are reminiscent of *rk *mutants, yet severe cuticular defects in 69B>rk RNAi adults result in legs which cannot support their body weight. The absence of mobility in these flies likely contributes to their death within 24 hours after eclosion. Expression of rk RNAi with C855a-GAL4 does not effect the ability of progeny to eclose (Figure 8D). However, all C855a>rk RNAi progeny show great difficulty walking and climbing, as manifested by dragging their metathoracic legs during locomotion. A closer inspection of their leg morphology revealed kinked metathoracic tarsi which phenocopy the legs of *rk^1^/Df *flies (data not shown). Unlike *rk^1^/Df *flies, C855a>rk RNAi flies are able to expand their wings. Somewhat surprisingly, these wings never become rigid, resulting in a sagging appearance (Figure 8D). We also analyzed UAS-rk RNAi expression with MJ33a-GAL4; these flies are healthy and indistinguishable from wild-type controls (data not shown).

**Figure 9:**
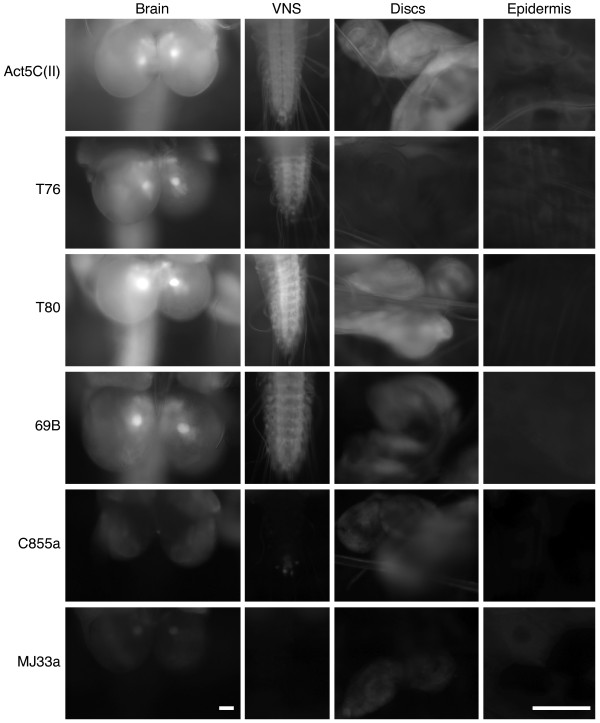
**Expression patterns of different GAL4 drivers in larval tissues**. The membrane-bound GFP reporter UAS-mCD8::GFP was expressed with different drivers to assay for strength of expression and pattern in CNS (brain), ventral nervous system (VNS), imaginal discs, and epidermal tissue. Compared to all other drivers, Act5C(II) expresses strongly in all tissue with solid patterns. T76 expresses strongly in CNS and epidermis. Expression in the imaginal discs is faint, but uniform. T80 expresses strongly in CNS and imaginal discs, but is absent from epidermal tissue. 69B expression is strong in CNS and imaginal discs. It can also be detected in a weak, very restricted pattern in the epidermal tissue. C855a expression is noticeably restricted in CNS and imaginal discs, as compared to Act5C(II). Expression is completely absent in the epidermis. MJ33a expression is restricted in the brain, extremely weak in the epidermis and discs, and absent from the VNS. For imaginal discs, examples from wing, haltere or metathoracic leg disc are shown. Epidermis refers to the epidermal tissue of the larval body wall.

The variability in RNAi phenotypes that we observed is likely due to tissue-specific *rk *requirements. To determine where rk RNAi expression results in mutant phenotypes, we expressed UAS-mCD8::GFP and analyzed GAL4 lines for their expression pattern and relative levels in the larval CNS, imaginal discs, and epidermis. In the CNS (brain and ventral nervous system) there is either a strong solid pattern, as in Act5C(II), T76, T80, and 69B, or expression is severely restricted as in C855a and MJ33a (Figure 9).

**Figure 10:**
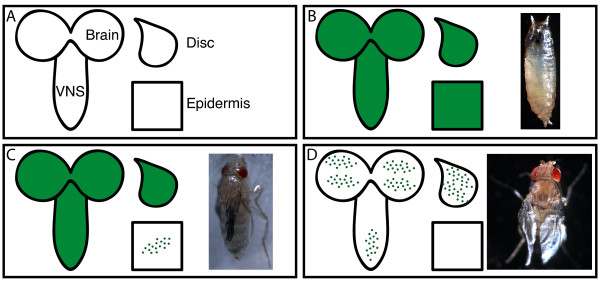
**Comparisons between GAL4 expression patterns and UAS-rk RNAi phenotypes**. (A) Key to the schematics. Assayed tissues are labeled as brain, VNS, imaginal discs and epidermis. (B) Correspondence of T76 expression pattern with T76>rk RNAi phenotype. Uniform expression in all tissues assayed appears to prevent the progression of prepupal development, resulting in small, untanned puparia. (C) Correspondence of 69B expression pattern with 69B>rk RNAi phenotype. Solid expression in brain, VNS, and imaginal discs, but weak pattern in epidermis, appears to result in flies with flaccid legs and unexpanded wings. (D) Correspondence of C855a expression pattern with C855a>rk RNAi phenotype. Extremely limited expression in all tissues assayed still results in adult flies whose wings cannot expand. The limited expression pattern likely allows *rk *expression required for wing expansion behaviors, but subsequent tanning of the wing is not possible, resulting in the sagging wing appearance. In the panels, solid green tissue symbolizes a uniform GFP pattern as observed in Figure 9, whereas the green punctae symbolize a restricted pattern of GFP expression.

The expression patterns of T76, 69B and C855a are summarized in Figure 10, along with their respective RNAi phenotypes. Among these drivers, T76 is unique in having strong, uniform expression in the larval epidermis (in addition to expression within CNS and weak expression in imaginal discs). This suggests that rk RNAi expression in (but not limited to) the epidermis adversely affects puparium (and possibly pupal cuticle) formation (Figure 10B). The epidermal expression pattern of 69B is weak and restricted to isolated patches, but there is strong solid expression in the imaginal discs (Figure 10C). Interestingly, we also observed abnormal leg shrugging in 69B>rk RNAi animals which fail to eclose. The eclosion defect may result from the inability to utilize the legs in extrication behaviors (see Discussion). In combination with the flaccid leg phenotype in eclosed 69B>rk RNAi flies, these observations implicate a requirement for *rk *in strengthening the cuticle of legs before eclosion. All assayed tissue patterns are more restricted in C855a-GAL4 (Figure 10D). Imaginal discs and CNS show highly restricted patterns, but no expression is observed in the epidermis. These patterns imply that the limited rk RNAi expression in CNS and imaginal discs permits wing expansion without subsequent tanning.

## Discussion

There is no question that the hormone bursicon, acting through its receptor rickets, is responsible for several post-eclosion events in *Drosophila*: deploying wing expansion behaviors [27], and plasticization and tanning of the new cuticle [1]. However, no cuticular tanning function earlier in development has yet been convincingly demonstrated. To our knowledge, ours is the first study in *Drosophila *to quantify the peripheral release of bursicon during the larval stage, and to experimentally manipulate targets of bursicon to investigate the role of this "tanning hormone" prior to eclosion.

To do so, we first wanted to confirm where bursicon is expressed and released in the periphery during development. While we were able to document bursicon release from larval type III boutons by immunocytochemistry, we also found this method to be difficult to perform quantitatively. The greatest obstacle to this approach is titrating the concentration of primary and secondary antibodies to 'biologically relevant' levels (as evinced by the smaller change in BURS-IR between DMH and DVP, compared to CCAP>ANF-EMD larvae). Instead, we favored *in vivo *measurements which exploit the UAS-ANF-EMD construct, which codes for the rat ANF prepropeptide tagged with the emerald variant of GFP [17]. Importantly, this ectopic neuropeptide also has no biological activity in *Drosophila*. The ANF-EMD marker has been used on numerous occasions to monitor vesicular release in *Drosophila *[28-33], and has become an effective method to study hormone release during an endogenously performed behavior [18-21]. Of these behavioral studies, one of the most convincing uses of UAS-ANF-EMD focused on its release from CCAP neurons in the ventral ganglion [18]. Preparations were compared at times before and after the 2^nd ^larval ecdysis, and the decrease in ANF-EMD fluorescence corresponded with a previously observed decrease in CCAP immunoreactivity between these same stages [34]. Thus measuring fluorescence dynamics in cells expressing UAS-ANF-EMD is a reliable method for studying the relationship between a behavior and the underlying hormonal release.

Our results indicating ANF-EMD release following larval ecdysis are consistent with a role for bursicon in tanning the new cuticle [9]. Strikingly, we also observed a significant amount of ANF-EMD release earlier, between the DMH and DVP stages. At the 2^nd ^larval ecdysis, the DMH stage can precede DVP (at which time ETH is first released) by up to 2 hours, with the end of ecdysis occurring approximately 35 minutes after DVP (see [22]).

Taken together, the BURS-IR and ANF-EMD fluorescence dynamics initiated between DMH and DVP stages represent bursicon release at the NMJ, preceding ETH release. This is in direct contrast to the current model of the neuroendocrine regulation of ecdysis, which places ETH release at the top of the sequence [35]. It is not clear if our observed pre-DVP bursicon release could be part of the actual neuroendocrine cascade that regulates ecdysis behaviors, or if it is released in parallel. One possibility is that this initial phase of bursicon release in larvae is used to tan the new mouthparts. However, we never observed un-tanned mouthparts in larvae expressing either burs RNAi or rk RNAi.

Another possibility is that our release data may include an unknown role for the BURS subunit. To detect bursicon, we used an antibody to the BURS subunit, although the active bursicon hormone is a heterodimer of both BURS and PBURS [7]. Furthermore, among the type III boutons that we sampled for fluorescence intensity are those that receive projections from neuromere T3, which express the BURS subunit but not PBURS [7]. It is currently unknown if BURS has any function in the absence of PBURS [cf. [7]], so we cannot rule out the possibility that our analyses include more than just heterodimeric bursicon release. Nevertheless, our results demonstrate that the type III boutons actively release vesicles at developmentally relevant time points. Future functional studies should be directed at deciphering the role of hormones secreted from type III boutons, especially just prior to the second larval ecdysis.

Following the demonstration that bursicon is released in *Drosophila *during development, we focused on its role at target tissues during development. To do so, we relied on the use of a transgenic RNAi construct to disrupt *rickets *expression in separate tissue domains. Similarly, a recent study in *Drosophila *also effectively used targeted RNAi against 'sex peptide receptor' to define specific neurons in females that are required for the behavioral response to 'sex peptide' [36]. In retrospect our transgenic approach was also necessary to sufficiently silence *rk *expression, relative to available *rk *mutants. Our analysis of *rk^1 ^*and *rk^4 ^*provides clear genetic evidence that they are not null mutants. By focusing on leg deformities (an aspect of the *rk *phenotype which shows incomplete penetrance in homozygous stocks), we were able to show that both *rk^1 ^*and *rk^4 ^*hemizygotes are afflicted with much more severe leg deformities. These results underscore the utility of RNAi in gene disruption studies, even when genetic mutants are available.

The ability to enhance the penetrance of deformed leg segments when *rk *alleles are hemizygous with the *Df(2L)BSC252 *deletion also suggests that some aspect of signal transduction is retained in *rk *mutants. Signal transduction in the rickets receptor activates PKA, through increasing cAMP levels [37]. Recently, post-eclosion tanning has been linked to phosphorylation of tyrosine hydroxylase (TH) by PKA, as a result of rickets activation [38]. A detectable TH activity profile in *rk^4 ^*pupae and adults follows the same pattern as TH activity in wild-type animals, yet at an attenuated level (see [38]). Although TH activity is an indirect measurement of rickets activation, we feel that the trend in TH activity shown by *rk^4 ^*pupae is in accordance with our view that *rk *alleles are not complete loss of function mutations.

By contrast, we currently do not know how the *rk *mutations could retain functionality, since they contain stop codons which should critically truncate the protein upstream of the transmembrane domain (*rk^1^*) or within the transmembrane domain (*rk^4^*) [5]. One possible scenario is that read-through of the stop codons is occurring at some level in these animals. The existence of this phenomenon has previously been demonstrated in *Drosophila*, in a rigorous analysis of read-through in the *Synapsin *gene [39]. To confirm a similar situation in *rk *mutants would require antibodies that recognize the rickets protein on either side of the stop codon. Regardless, a more thorough analysis of the function of rickets will have to await a mutation which exclusively removes its entire coding sequence.

The determination that *rk *mutants are not nulls validates our use of rk RNAi, which effectively reduces *rk *transcript levels, and is lethal when expressed ubiquitously. Conveniently, the rk RNAi phenotypes we observed with more selective GAL4 lines (including imaginal disc or epidermal patterns) are consistent with a role for bursicon signaling at the epidermis to mediate cuticle tanning (cf. [38]). Even so, our results were still unexpected. As in some Act5C>rk RNAi pupae, T76>rk RNAi have a terminal phenotype with small, soft, misshapen puparia. We believe this is due to disrupted *rk *expression in the epidermis. T76>rk RNAi prepupae never advance to a point where a distinct pupa can be observed, which also suggests that *rk *expression in the epidermis may be important in formation of the developing pupal cuticle. The soft, flexible puparium observed in T76>rk RNAi is especially intriguing, since bursicon signaling has never before been directly linked to pupariation. Hardening of the puparium in Diptera is instead believed to be regulated by another peptide hormone, the "puparium tanning factor" [40-42], which has been identified as a pyrokinin in the grey flesh fly (*Sarcophaga bullata*) [42].

Two genotypes resulted in trapped pharate adults that were unable to eclose: T80>rk RNAi and 69B>rk RNAi. Fortuitously, we observed 69B>rk RNAi flies attempting to eclose, and a small fraction of these animals were successful. Based on the GAL4 expression patterns in imaginal discs of T80 and 69B, we hypothesize that *rk *is required to sclerotize the cuticle, and in particular the legs, during the pharate adult stage. The resulting legs of 69B>rk RNAi flies which successfully eclose are extremely flaccid and unable to support the weight of the fly (Figure 8C). Although leg movements have not been shown to be required for eclosion (cf. [43]), we believe that the failure to eclose in the majority of 69B>rk RNAi flies may be due to their flaccid legs. Mature 69B>rk RNAi pharate adults that failed to eclose exhibited abnormal leg shifting movements within the puparium. These consisted of coxal shrugging movements, accompanied by displacement of the femora with slipping movements. Previous results in the tobacco hornworm *Manduca sexta *[44] and also in *Sarcophaga bullata *[45] indicate that restraint of the legs are important stimuli to elicit extrication from the pupal case. Since the legs are defective in 69B>rk RNAi animals, the absence of this crucial leg restraint signal may lead to the observed failure at eclosion.

We also showed that a more restricted GAL4 pattern can result in less severe RNAi phenotypes. This is exemplified by C855a>rk RNAi flies, which succeed in expanding their wings following eclosion. Curiously the wings never harden, giving all C855a>rk RNAi wings a unique sagging appearance. Examination of the C855a expression pattern revealed limited CNS expression, especially in the VNS, whereas bursicon signaling within the CNS is required to deploy wing expansion behaviors [27]. Perhaps RNAi can be used in future studies to determine the CNS targets in this pathway with greater accuracy. To account for the "sagging wing" phenotype, the reduced expression pattern in C855a wing discs (relative to T80 and 69B) is evidently sufficient to disrupt tanning in the wings of C855a>rk RNAi. Finally, MJ33a>rk RNAi flies appeared normal, and from this we assume that their greatly restricted expression patterns in CNS, imaginal discs and epidermis are insufficient for disrupting *rk *expression.

The peptide hormones which regulate ecdysis may interact in complicated ways which do not fit into a linear model of causation (cf. [34]). Here, we have shown that rickets activity, traditionally placed after eclosion, also has unexpected roles in tanning the puparium and strengthening pharate adult cuticle. These observations would not have been possible without silencing *rk *expression in tissues where it is required to respond to bursicon. It is noteworthy here to mention a recent study which examined the roles of all ecydsis-related peptide hormones and their receptors in the flour beetle *Tribolium castaneum*, using systemic RNAi [46]. Apart from effecting cuticular tanning, this study revealed that injections of *burs/pburs *or *rk *RNAi into pharate pupae resulted in diminished strength of contractions at pre-ecdysis. Our own study clearly indicates that rickets plays a central role prior to eclosion.

## Conclusions

Our results redefine bursicon signaling as an essential pathway during *Drosophila *development. We present new evidence during larval ecdysis that bursicon secretion precedes the canonical ecydsis cascade of neuropeptide regulation. Furthermore, ubiquitous inhibition of bursicon expression results in 100% lethality by the pharate adult stage, thus preventing eclosion. Finally, rickets expression in the epidermis/imaginal discs is crucial for *Drosophila *development, especially during the pupal stage. These results indicate that bursicon activity is not confined to post-eclosion development in *Drosophila*, as previously postulated.

## Methods

### Fly stocks

All stocks were reared on standard media at 25°C in 12 h:12 h LD cycle. Where required, larvae were reared on grape juice plates (3% agar) augmented with yeast to improve their development. Except where mentioned, all stocks are available from the Bloomington Drosophila Stock Center (accompanied by stock number). The stock *w^1118 ^*(#6326) was used as the wild-type strain. A stock of CCAP-GAL4 flies (gift of John Ewer) was used to drive expression in type III boutons of UAS-ANF-EMD (#7001; [17]). Recombinants of elav-GAL4 with UAS-ANF-EMD were used as control animals for the CCAP>ANF-EMD release experiments. In most instances, ubiquitous GAL4 expression was attained by an Act5C-GAL4 insertion on the 3^rd ^chromosome (#3954), although a 2^nd ^chromosome insertion of Act5C-GAL4 (#4414) was used as a positive control and for UAS-mCD8::GFP (#5137) expression. For clarity, this additional GAL4 stock is referred to as Act5C-GAL4(II) in the text. We used *y^1 ^v^1^; P{TRiP.JF02260}attP2 *(#26719) for UAS-burs RNAi, and *w^1118^; P{GD14383}v29931 *(Vienna *Drosophila *RNAi Center) for UAS-rk RNAi. For expression in tissue subsets which include imaginal discs and/or epidermis, we used the following GAL4 drivers: T76 (#6995), T80 (#1878), 69B (#1774), C855a (#6990), and MJ33a (#6992). Additional GAL4 drivers used in this study include: c929 [47], 386 [48], n-syb (gift from Julie Simpson), 36 y (gift from Paul Taghert), r4 (gift from Jae Park), and FB (gift from Thomas Neufeld). For genetic analysis of the *rickets *mutations, we used the classic alleles *rk^1 ^cn^1 ^bw^1 ^*(#3589) (referred to as *rk^1 ^*in the text) and *rk^4 ^*(#3590), in addition to the deficiency *Df(2L)BSC252/CyO *(#23152), which deletes the *rickets *gene in addition to adjacent genomic regions [23]. A stock of *Df(2L)BSC252 *balanced with *CyO, ActGFP *(#4533) was also used in some cases, to facilitate selection of progeny bearing the deletion. To document phenotypes, whole-animal bright field images were taken on a Leica MZFLIII microscope and saved with Leica IM50 (version 1.20) software.

### Immunocytochemistry

Wandering 3^rd ^instar larvae were dissected in cold Ca^2+^-free HL3 [49] and filleted. For vesicle membrane protein experiments, preparations were fixed for one hour at room temperature in Bouin's fixative. Otherwise, all other preparations were fixed for 30 minutes at room temperature in 4% paraformaldehyde. Fillets were thoroughly washed in PBSTx (PBS + Tween + 0.3% Triton X-100), and incubated in 20% normal donkey serum for 1 hour. Tissues were then incubated in primary antibody overnight at 4°C, quickly rinsed and then washed 3 × 15 minutes in PBSTx. Subsequently, preparations were incubated for 3 hours in secondary antibody at room temperature, thoroughly rinsed and washed in PBSTx, and finally mounted on slides in Vectashield (Vector Laboratories) and stored in the dark at 4°C. The one exception was that larval tracheae processed for ETH-IR were mounted on poly-lysine-D - coated cover slips, dehydrated through an ethanol series into xylene, and mounted in DPX mountant (EMS). Primary antibodies used include rabbit anti-BURS (bursicon) (1:5000, or 1:20,000 for bursicon release analysis) (a generous gift from Benjamin White), rat anti-R29 (N-SYB) (1:200; a generous gift from Hugo Bellen), mouse anti-DCSP2 (CSP) (1:100; Developmental Studies Hybridoma Bank), goat anti-HRP (1:400; Jackson ImmunoResearch Laboratories), rabbit anti-CCAP (1:5000; a generous gift from John Ewer), and rabbit anti-ETH (1:180,000; a generous gift from John Ewer). For secondary antibodies, the relevant species was used with excitations of either 488 nm or 594 nm (1:1000; Invitrogen). For fluorescence imaging, histological preparations were viewed on a Nikon Eclipse E600FN microscope at 40×. Images were collected with a SPOT2 camera (Diagnostic Instruments, Inc.) as 8-bit monochrome with the SPOT32 software (version 2.2).

### Fluorescence intensity measurements

The GFP-tagged 'atrial natriuretic factor' reporter, UAS-ANF-EMD, was expressed with CCAP-GAL4 to monitor *in vivo *neuropeptide release from bursicon-releasing CCAP neurons in CCAP>ANF-EMD progeny. Images from live fluorescent tissue were collected as described above for histological preparations. To measure ANF-EMD release from type III boutons in larvae, we chose six developmental stages that include the 2^nd ^larval ecdysis. To obtain larvae approaching this ecdysis, it was necessary to collect CCAP>ANF-EMD embryos on grape juice plates with 3% agar, supplemented with yeast. The recognition of these stages is described in the Results. Staged animals were dissected in Ca^2+^-free HL3 saline with a dorsal - longitudinal incision and splayed open with pins on magnetic plates. For each stage a distinct set of four animals was selected and all visible type III boutons were photographed with the same exposure setting. Since CCAP-GAL4-expressing boutons occur bilaterally in NMJ 12 of the T3-A4 segments, the maximum number of type III boutons that can be analyzed per animal is 5 pairs, although we were not always able to visualize this maximum number. With ImageJ software, we empirically determined a threshold at which we could select type III bouton area in animals preceding ecdysis (at the 'double mouth hook stage'), and with this threshold value we measured mean pixel value in type III boutons at all stages. To calculate average fluorescence intensity, we multiplied the area (selected by the threshold) and mean pixel value, and took the average of these values for each stage. Comparisons between stages to determine percent release were calculated as [fluorescence intensity_0_]-[fluorescence intensity_1_]/[fluorescence intensity_0_]. Error bars represent the standard error of the mean (SEM). Single-factor ANOVA tests were also performed to determine if the fluorescence intensity from adjacent stages differed significantly from each other. As a control against large-scale vesicular release at the NMJ, the pan-neural elav-GAL4 driver was used to express ANF-EMD at all NMJs. Confirmation of bursicon release by BURS-IR was performed identically.

Similar procedures were performed to analyze pupal ecdysis. CCAP>ANF-EMD pupae were harvested from the culture vial walls at the following stages of metamorphosis: P3, P4(i), P4(ii), and P5(i). These stages were identified according to standard morphological characters associated with their development [50]. We assayed stages from initial puparium formation through the completion of pupal ecdysis. For each time point, all observable type III boutons were measured from two distinct pupae. Tracking release of the ANF-EMD reporter from discrete animals for each time point has been performed with success in the past (cf. [18]).

### Histological preparation and analysis of legs

Adult metathoracic legs were surgically dissected, cleared by overnight incubation in 10% KOH at room temperature, dehydrated through an ethanol series, and mounted in Euparal (BioQuip Products, Inc.). Leg preparations were subsequently viewed on a Nikon Eclipse E600FN microscope at 10× and photographed with a SPOT2 camera (Diagnostic Instruments, Inc.) as 8-bit monochrome with the SPOT32 software (version 2.2).

For convenience, we narrowed our phenotypic observations to the metathoracic tarsi, although it was not uncommon for additional leg segments to also show deformities. We classified tarsal phenotypes into three categories of increasing severity and scored them as either resembling wild-type; exhibiting a kink in the 1^st ^tarsal segment (T1); or exhibiting a bulbous 2^nd ^tarsal segment (T2) with rotated distal tarsal segments, in addition to a T1 kink.

### Imaging

Equal exposure settings were maintained while comparing experimental and control samples, or comparing samples across different time points. All images were cropped and monochrome images were given their color identities with Adobe Photoshop CS (version 8.0). Figures were finalized with Adobe Illustrator CS (version 11.0).

### Assay of rickets transcript levels by reverse-transcription PCR

RT-PCR was used to assay *rickets *levels in pupae with ubiquitous expression of rk RNAi (Act5C>rk RNAi) and its control, UAS-rk RNAi pupae. For a loading control, levels of *RpS26 *were also compared between the two genotypes. Pupae of each genotype were homogenized in TRIZOL Reagent (Invitrogen) to isolate total RNA (following the supplied protocol). We reverse-transcribed 5 μg of total RNA with oligo dT, according to the manufacturer's instructions with SuperScript III enzyme (Invitrogen). For amplification, we used 1 μl of cDNA per 50 μl reaction with DYNAzyme EXT (Finnzymes), according to the manufacturer's instructions. Primers specific to the rickets gene were used to amplify cDNA templates. For *rk*, the forward primer sequence was 5'-CATACACAAGGAAGCCTTTTCC-3' and the reverse primer sequence was 5'-TTAATAGCCGTCTCCCAAGG-3'. For *RpS26*, the forward primer sequence was 5'-CCCGAAACGTGAACACACGCGG-3' and the reverse primer sequence was 5'-GGCCGCGATTGTGCTTGTTGCGTCC-3'. The following program was used to amplify the products. The initialization step was 1 cycle of 94°C for 2 minutes; followed by 35 cycles of 94°C for 15 seconds, 55°C for 15 seconds, 72°C for 1 minute; with a final elongation step of 1 cycle of 72°C for 5 minutes. PCR products were analyzed by agarose gel electrophoresis (1.5% agarose in 1× TAE) and visualized with ethidium bromide staining under UV fluorescence.

## Competing interests

The authors declare that they have no competing interests.

## Authors' contributions

BJL performed most experiments, while DLD performed the RT-PCR, in addition to providing material support to BJL. Both BJL and DLD contributed to the conception of this study, as well as writing of the manuscript. Both authors read and approved the final manuscript.

## Supplementary Material

Additional file 1 **A decrease in BURS-IR precedes any change in ETH-IR at the second larval ecdysis**. Paired (A) ETH-IR and (B) BURS-IR from the same larva at DMH, prior to ecdysis. Paired (C) ETH-IR and (D) BURS-IR from an additional larva at DVP, upon the initiation of ecdysis. (E) Relative average fluorescence intensities for BURS-IR and ETH-IR, at DMH and DVP. The changes in fluorescence intensity indicate bursicon release during this time period, while no release was apparent for ETH in the same time interval. (A-D) are characteristic images represented in the analysis (E). Error bars indicate +/- SEM. Scale bar = 10 μm. Click here for file
