# Supplementary figures and images for: The essential role of bursicon during Drosophila development
Source: BMC Dev Biol. 2010 Aug 31;10:92. doi: 10.1186/1471-213X-10-92 (PMC2942807; doi:10.1186/1471-213X-10-92)

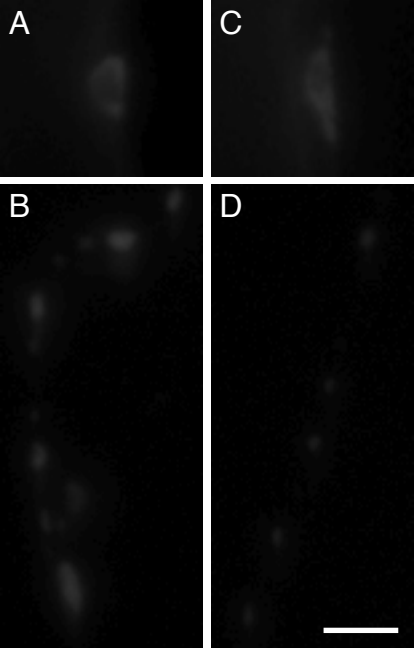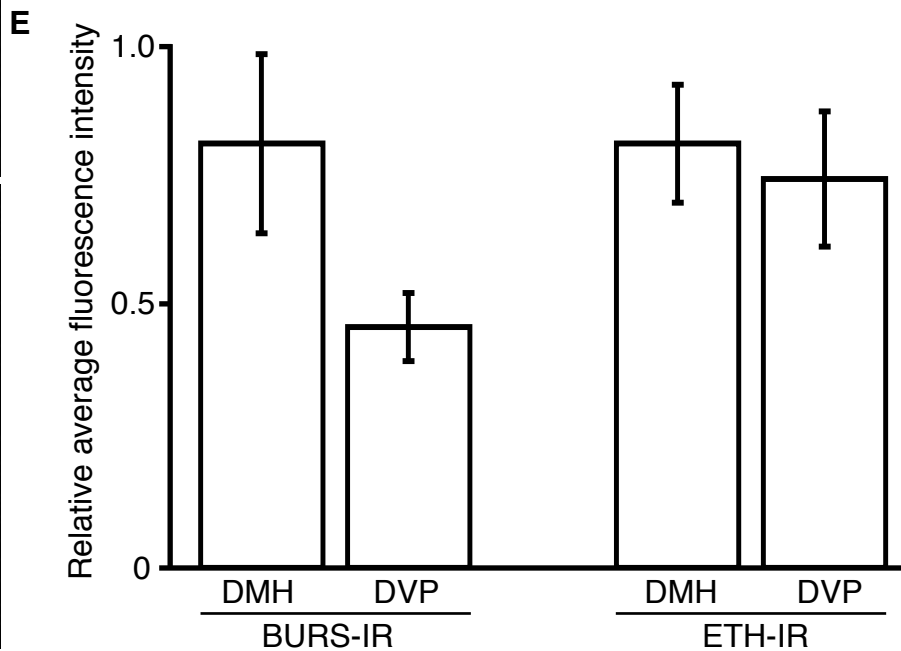

Supplement: Additional file 1 — A decrease in BURS-IR precedes any change in ETH-IR at the second larval ecdysis. Paired (A) ETH-IR and (B) BURS-IR from the same larva at DMH, prior to ecdysis. Paired (C) ETH-IR and (D) BURS-IR from an additional larva at DVP, upon the initiation of ecdysis. (E) Relative average fluorescence intensities for BURS-IR and ETH-IR, at DMH and DVP. The changes in fluorescence intensity indicate bursicon release during this time period, while no release was apparent for ETH in the same time interval. (A-D) are characteristic images represented in the analysis (E). Error bars indicate +/- SEM. Scale bar = 10 μm. [file 1471-213X-10-92-S1.PDF]
